# Supplementary material for: Splice-Junction-Based Mapping of Alternative Isoforms in the Human Proteome
Source: Cell Rep. Author manuscript; Available in PMC 2020 Jan 15. (PMC6961840; doi:10.1016/j.celrep.2019.11.026)
Supplement: 2 [file NIHMS1546469-supplement-2.zip › DF1/PXD010154/Esophagus/Esophagus_26_QARS_TQWNPAYWTCQSHQFQLWLCQGQ.pdf]

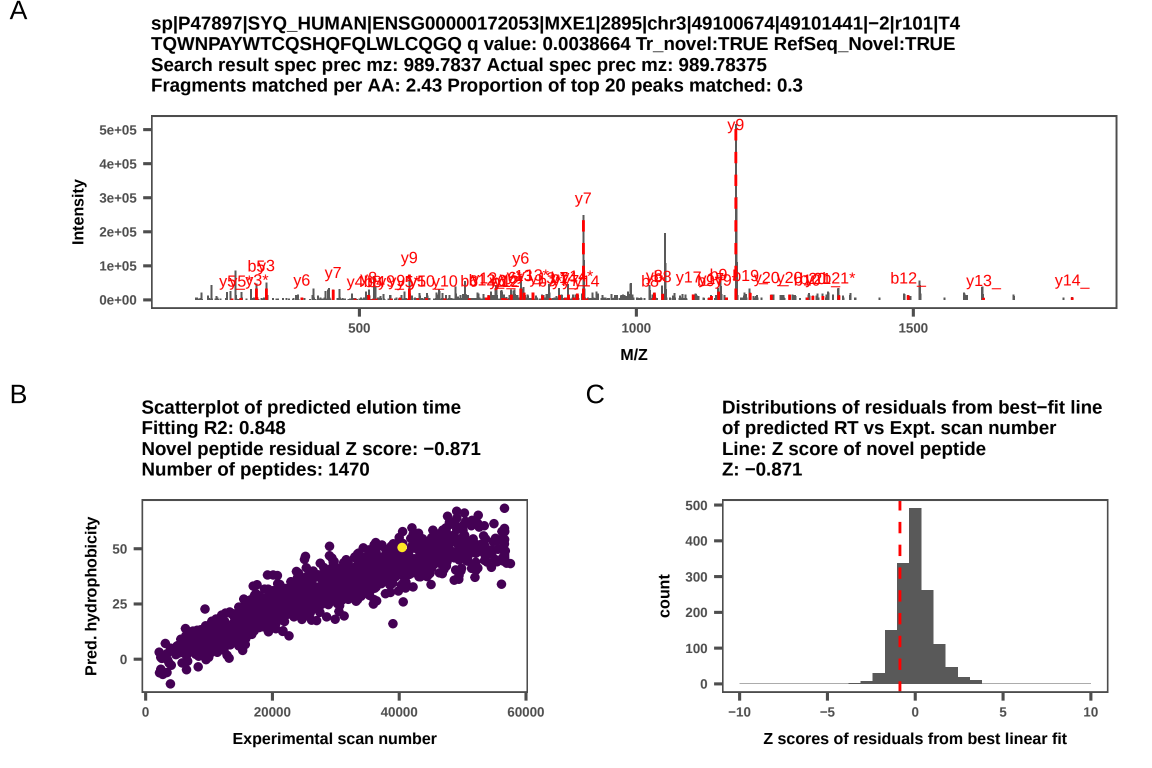
A
sp|P47897|SYQ_HUMAN|ENSG00000172053|MXE1|2895|chr3|49100674|49101441|−2|r101|T4
TQWNPAYWTCQSHQFQLWLCQGQ q value: 0.0038664 Tr_novel:TRUE RefSeq_Novel:TRUE
Search result spec prec mz: 989.7837 Actual spec prec mz: 989.78375
Fragments matched per AA: 2.43 Proportion of top 20 peaks matched: 0.3
Intensity
5e+05
4e+05
3e+05
2e+05
1e+05
0e+00
y9
y7
y9
y6
b53
y6
y7
b12_
y13_
y14_
500
1000
1500
M/Z
B
Scatterplot of predicted elution time
Fitting R2: 0.848
Novel peptide residual Z score: −0.871
Number of peptides: 1470
Pred. hydrophobicity
50
25
0
0
20000
40000
60000
Experimental scan number
C
Distributions of residuals from best−fit line
of predicted RT vs Expt. scan number
Line: Z score of novel peptide
Z: −0.871
count
500
400
300
200
100
0
−10
−5
0
5
10
Z scores of residuals from best linear fit
